# Seizure control via pH manipulation: a phase II double-blind randomised controlled trial of inhaled carbogen as adjunctive treatment of paediatric convulsive status epilepticus (*C*a*r*bog*e*n for *S*tatus Epilepticus in *C*hildr*en T*rial (CRESCENT))

**DOI:** 10.1186/s13063-024-08188-5

**Published:** 2024-05-29

**Authors:** Rob Forsyth, Maria Allen, Emma Bedson, Alice Downes, Chris Gough, Stuart Hartshorn, Kelly Lawton, Mark D. Lyttle, Shrouk Messahel, Niall Mullen, Joseph Raper, Anna Rosala-Harris, Leigh Taggart, Jason Urron, Emily Walton, Carrol Gamble, on behalf of the Paediatric Emergency Research in the United Kingdom and Ireland (PERUKI) collaborative, Meriel Tolhurst-Cleaver, Sarah Siner, Anandi Singh, Elizabeth Ledger, Thomas Harris, Rachel Amos, Alice J. Downes, Sally Price, Maria Mikhail, Noor Alhashimi

**Affiliations:** 1 Newcastle Upon Tyne Hospitals NHS Foundation Trust, Newcastle Upon Tyne, UK; 2 Newcastle University, Newcastle Upon Tyne, UK; 3 Liverpool Clinical Trials Centre, University of Liverpool, Liverpool, UK; 4 Leeds Teaching Hospitals NHS Trust, Leeds, UK; 5 Nottingham University Hospitals NHS Trust, Nottingham, UK; 6 Birmingham Children’s Hospital NHS Trust, Birmingham, UK; 7 Emergency Department, Bristol Royal Hospital for Children, Bristol, UK; 8 Research in Emergency Care Avon Collaborative Hub (REACH), University of the West of England, Bristol, UK; 9 Alder Hey Children’s NHS Foundation Trust, Liverpool, UK; 10 Sunderland and South Tyneside NHS Foundation Trust, Sunderland, UK; 11 University Hospitals Sussex NHS Foundation Trust, Worthing, UK; 12 Newcastle upon Tyne, UK

**Keywords:** Convulsive status epilepticus, Acid–base, pH, Respiratory acidosis, Paediatrics, Deferred consent

## Abstract

**Background:**

Paediatric convulsive status epilepticus is the most common neurological emergency presenting to emergency departments. Risks of resultant neurological morbidity and mortality increase with seizure duration. If the seizure fails to stop within defined time-windows, standard care follows an algorithm of stepwise escalation to more intensive treatments, ultimately resorting to induction of general anaesthesia and ventilation. Additionally, ventilatory support may also be required to treat respiratory depression, a common unwanted effect of treatment. There is strong pre-clinical evidence that pH (acid–base balance) is an important determinant of seizure commencement and cessation, with seizures tending to start under alkaline conditions and terminate under acidic conditions. These mechanisms may be particularly important in febrile status epilepticus: prolonged fever-related seizures which predominantly affect very young children. This trial will assess whether imposition of mild respiratory acidosis by manipulation of inhaled medical gas improves response rates to first-line medical treatment.

**Methods:**

A double-blind, placebo-controlled trial of pH manipulation as an adjunct to standard medical treatment of convulsive status epilepticus in children. The control arm receives standard medical management whilst inhaling 100% oxygen; the active arm receives standard medical management whilst inhaling a commercially available mixture of 95% oxygen, 5% carbon dioxide known as ‘carbogen’.

Due to the urgent need to treat the seizure, deferred consent is used.

The primary outcome is success of first-line treatment in seizure cessation. Planned subgroup analyses will be undertaken for febrile and non-febrile seizures. Secondary outcomes include rates of induction of general anaesthesia, admission to intensive care, adverse events, and 30-day mortality.

**Discussion:**

If safe and effective 95% oxygen, 5% carbon dioxide may be an important adjunct in the management of convulsive status epilepticus with potential for pre-hospital use by paramedics, families, and school staff.

**Trial registration:**

EudraCT: 2021-005367-49. CTA: 17136/0300/001. ISRCTN: 52731862. Registered on July 2022.

## Administrative information

Note: the numbers in curly brackets in this protocol refer to SPIRIT checklist item numbers. The order of the items has been modified to group similar items (see http://www.equator-network.org/reporting-guidelines/spirit-2013-statement-defining-standard-protocol-items-for-clinical-trials/).

**Table:** 

Title {1}	*C*a*r*bog*e*n for *S*tatus Epilepticus in *C*hildr*en T*rial (CRESCENT) Seizure control via pH manipulation: a phase II double blind RCT of inhaled carbogen as adjunctive treatment of paediatric convulsive status epilepticus
Trial registration {2a and 2b}.	EudraCT: 2021–005367-49 CTA: 17136/0300/001 ISRCTN: 52731862 (registered 27/7/2022)
Protocol version {3}	V3.0 17/11/2022
Funding {4}	National Institute of Health Research (UK) Efficacy and Mechanism Evaluation Programme: Funder Ref: NIHR 129875
Author details {5a}	Rob Forsyth^1,2^, Maria Allen^1^, Emma Bedson^3^, Alice Downes^4^, Chris Gough^5^, Stuart Hartshorn^6^, Kelly Lawton, Mark D. Lyttle^7,8^, Shrouk Messahel^9^, Niall Mullen^10^, Joseph Raper^1^, Anna Rosala-Harris^3^, Leigh Taggart^3^, Jason Urron^1^, Emily Walton^11^, Carrol Gamble^3^ ^1^Newcastle upon Tyne Hospitals NHS Foundation Trust ^2^Newcastle University ^3^Liverpool Clinical Trials Centre, University of Liverpool ^4^Alice Downes, Leeds Teaching Hospitals NHS Trust ^5^Nottingham University Hospitals NHS Trust ^6^Birmingham Children’s Hospital NHS Trust ^7^Emergency Department, Bristol Royal Hospital for Children, Bristol, UK ^8^Research in Emergency Care Avon Collaborative Hub (REACH), University of the West of England, Bristol, UK ^9^Alder Hey Children’s NHS Foundation Trust ^10^Sunderland and South Tyneside NHS Foundation Trust ^11^University Hospitals Sussex NHS Foundation Trust
Name and contact information for the trial sponsor {5b}	Rebecca Errington, The Newcastle upon Tyne Hospitals NHS Foundation Trust, Freeman Rd, High Heaton, Newcastle upon Tyne NE7 7DN, United Kingdom
Role of sponsor {5c}	The funder sought independent peer review before funding but has no role in study design, data collection and analysis, decision to publish, or preparation of manuscripts. The sponsor is the Newcastle upon Tyne Hospitals NHS Foundation Trust (NUTH) and is legally responsible for the study. The sponsor has delegated duties for study design; data collection, analysis, interpretation; and publication of results to the CI and LCTC who undertake these activities in co-ordination with members of the TMG, TSC and IDSMC. These oversight committees will ensure the timely publication and accurate interpretation of the study results

## Introduction

### Background and rationale {6a}

Convulsive status epilepticus (CSE) is the most common neurological emergency in children, affecting 20 per 100,000 children per year [1]. This equates to 4000 CSE events in England and Wales annually, of which half will be in children who have never previously had a seizure. The first episode of CSE is a terrifying experience for parents, many of whom believe their child is dying [2]. The morbidity of CSE relates to cause and duration; despite improved pre-hospital care, mortality remains 3% [1, 3]. Current treatment for CSE follows a standardised stepwise algorithm. Benzodiazepines (midazolam, lorazepam or diazepam) are given at most twice parenterally (typically buccally, intravenously or rectally: increasingly one or both doses are administered pre-hospital) with escalation to second-line treatments (traditionally phenytoin or phenobarbital, increasingly levetiracetam) if seizures have not stopped. If these fail, general anaesthesia is given via rapid sequence induction (RSI) and the child is admitted to an intensive care unit (ICU) [4]. Children with previous episodes of CSE will sometimes have a personalised seizure management plan, which may use alternative first-line medications including paraldehyde, however, second-line and subsequent management tends to converge on standard practice. Parenteral benzodiazepines become less effective with increasing CSE duration: suggested mechanisms include internalisation of GABA receptors [5] or the loss of GABA-A receptor conductance and chloride-extrusion function [6]. There is therefore a growing practice of supplying buccal midazolam for pre-hospital use, but repeated benzodiazepine administration has a high risk of respiratory depression and contributes to ICU admission [7].

Lyttle et al*.* recently successfully completed EcLiPSe, a randomised controlled trial (RCT) in UK paediatric emergency departments (EDs) comparing phenytoin and levetiracetam as second-line CSE treatments in children who failed to respond to a second dose of benzodiazepine [8]. CRESCENT builds on the success of EcLiPSe, addressing the earlier, first stage of the CSE algorithm, aiming to improve the efficacy of first-line benzodiazepines, shorten duration of CSE, and reduce the need for second-line interventions, CSE-related morbidity, and hospital and ICU admission rates. CRESCENT draws on EcLiPSe for relevant aspects of its design.

Extensive pre-clinical and pilot data suggest that mild acidosis promotes seizure termination [9, 10]. Mild physiological acidosis can be achieved by replacing the oxygen typically delivered during management of CSE with 95% oxygen, 5% carbon dioxide, a medical gas mixture known as carbogen, which still provides oxygen in large excess. We see carbogen as a simple potential enhancement of current best practice, with the possibility of improved response to first-line treatments, a reduction in repeated administration of benzodiazepines, and thus treatment-related morbidity. In contrast, slightly increased circulating carbon dioxide levels may provide a modest stimulation to respiratory drive. Rebreathing masks that recirculate exhaled air would be an alternative means of achieving respiratory acidosis, although the magnitude would be hard to control in the context of seizures affecting minute-volume. In addition, they would not deliver supplemental oxygen. Carbogen is cheap, easy to store, and non-sedating; if shown to be safe and effective, this could in principle be delivered in pre-hospital settings by families, teachers, and paramedics.

Brain pH is 0.3 units more acidic than blood [11]: this compartmentation implies neurophysiological importance [12]. Several lines of evidence indicate a role for brain pH in seizure onset and termination. Respiratory alkalosis due to hyperventilation is a reliable trigger of seizures in childhood absence epilepsy [13]. Fever-induced hyperventilation may contribute to the common paediatric phenomenon of febrile convulsion [14, 15]. Intentional induction of mild respiratory alkalosis increases seizure frequency and duration in the context of electroconvulsive therapy [16, 17]. Mixed metabolic and respiratory alkalosis also appears important in the genesis of seizures in neonatal hypoxic ischaemic encephalopathy (birth asphyxia) [18].

Precisely how pH shifts affect seizure propagation remains incompletely understood but a role for acid-sensing ion channel 1a has been suggested [19]. Intracellular acidification achieved by increasing the proportion of inspired carbon dioxide (F_i_CO_2_) terminates hypomagnesemic [20] and bicuculline-induced [9] seizures in rodents and macaques. Seizure inhibition in a rodent audiogenic seizure model has been reported with F_i_CO_2_ of 5–15%. The anticonvulsant effects of acetazolamide (a carbonic anhydrase inhibitor) are at least in part due to the resulting increase in p_a_CO_2_ [21]. Carbonic anhydrase inhibition is also part of the mechanism of action of several established epilepsy medications including topiramate and zonisamide. A degree of seizure-related hypoventilation is seen even in focal seizures [22], and it is possible that respiratory and/or metabolic acidosis may contribute to ‘spontaneous’ seizure termination, although other processes are probably also important [23].

Carbogen inhalation results in a maintained increase in p_a_CO_2_ [24], even if buffering leads to secondary metabolic alkalosis and normalisation of the arterial pH. This affects the passive diffusion of CO_2_ out of the brain thus increasing intracerebral pH.^31^P magnetic resonance spectroscopy (MRS) confirms that 5% carbogen inhalation by healthy human volunteers achieves a sustained fall in brain intracellular pH [11]. Acquisition of the MRS data in this study took 20 min, confirming the sustained nature of the ∆pH and again the independence of intracerebral pH from any peripheral buffering. Direct current-EEG analysis in volunteers indicates near instantaneous onset of carbogen effects on brain neurophysiology that are again sustained despite rebuffering of blood pH [24, 25]. Carbogen-induced seizure termination in rodents is associated with cerebral extracellular ∆pH (measured continuously in real time by implanted electrode) of 0.05–0.1 units that persists for tens of minutes [14] despite presumed peripheral buffering (although peripheral pH was not examined in this study).

### Summary of rationale

Manipulation of brain pH may be a neglected approach to termination of CSE, particularly in febrile CSE which is responsible for a high proportion of paediatric CSE. Carbogen provides a straightforward means of manipulating brain pH in the context of CSE. Adding carbogen to the current management of CSE may be a useful adjunctive treatment, improving first-line treatment response rates.

### Objectives {7}

The objectives are to ascertain (i) the effectiveness of carbogen in enhancing response rates of conventional first-line treatments of CSE and (ii) its safety in this context.

### Trial design {8}

CRESCENT is a randomised, placebo-controlled, double-blind multicentre superiority trial.

The trial will be randomised in a 1:1 ratio with allocation concealed using identical, sequentially numbered individual gas canisters supplied specifically for the use of CRESCENT. Participants and clinicians are blind to the medical gas received. Participants will be analysed within the group they were randomly assigned in accordance with the gas canister utilised.

## Methods: participants, interventions, and outcomes

### Study setting {9}

Participants will be identified and recruited from paediatric Emergency Departments in England. Eight sites (a combination of tertiary (regional) and secondary (district) level units) have been opened initially, with funding for an additional four sites secured, if required (list of participating sites available at https://www.crescent-trial.org.uk).

### Eligibility criteria {10}

Patients eligible for the trial must fulfil all the following at randomisation:

1. Presenting to the paediatric (not adult) emergency department (ED) of a participating site
2. Exhibiting signs of convulsive status epilepticus (CSE) (i.e. ongoing generalised tonic–clonic, generalised clonic, or focal clonic convulsive seizure activity)
3. Requiring, in the view of the treating clinician, emergency treatment either according to standard Advanced Paediatric Life Support (APLS) guidelines or the child’s personalised rescue care plan

Personalised rescue care plans are defined as CSE treatment plans that have been individualised and adapted in light of prior experience of response to standard APLS treatment protocols. Personalised plans occasionally make use of paraldehyde (not part of standard APLS protocols); these children remain eligible for CRESCENT, and paraldehyde will be considered a first-line medication.

A patient meeting any of the criteria listed below at baseline will be excluded from study participation:

1. Known to have been previously enrolled in the CRESCENT trial
2. Infantile spasms (West syndrome)
3. Non-epileptic seizure (‘pseudo status epilepticus’)
4. Tonic posturing due to suspected brain herniation
5. Already received phenytoin, levetiracetam, phenobarbital, or valproate as part of the management of this current episode of status epilepticus

Known or suspected pregnancy is not an exclusion criterion.

Eligibility will be assessed by an appropriately qualified healthcare professional with the experience to diagnose CSE, consider and exclude differential diagnoses, and initiate emergency treatment.

### Who will take informed consent? {26a}

As CSE is a medical emergency a deferred consent model will be used, with consent sought once the child’s clinical condition has stabilised. This approach has been successfully used in previous paediatric ED trials [8, 26]. A research nurse or other designated member of the research team will approach the person with parental responsibility/representative/patient to seek consent as soon as is reasonable based on the patient’s clinical condition, based on the opinion of the clinical team (ideally within 24 h of randomisation). In situations where a child has been transferred or discharged before it was possible to approach for face-to-face consent, consent will be sought remotely: the family will be informed of their child’s participation by telephone, an information booklet will be posted and they will be invited to return a consent form by post (in cases of non-response one follow-up approach is permitted). A separate process has been developed for the unlikely event of a child dying of complications of their CSE before it was possible to approach for consent.

### Additional consent provisions for collection and use of participant data and biological specimens {26b}

Anonymised data will be transmitted to the Liverpool Clinical Trials Centre (LCTC) for all participants to allow monitoring of safety in accordance with the Medicines for Human Use (Clinical Trials) Regulations 2004 as amended. Personal identifiable data will only be transmitted to LCTC where written informed consent has been obtained.

## Interventions

### Explanation for the choice of comparators {6b}

Standard of care is 100% oxygen, usually administered via the ED’s fixed, piped oxygen supply infrastructure. 100% oxygen is the comparator for the trial administered by individual gas cannisters and non-rebreather single-use standard hospital face mask to support blinding.

### Intervention description {11a}

Inhalation of 100% oxygen or c carbogen (5% CO_2_:95% O_2_) delivered at 15 l/min via a non-rebreather single-use standard hospital face mask, for the first 10 min of treatment, was performed.

The high flow rate is to minimise the risk of dilution by entrainment of room air during inspiration. To maintain blinding in the trial, both the comparator and the investigational medical product (IMP; 5%CO_2_: 95%O_2_) are delivered in identically overpainted, small, CD size, medical gas cylinders.

After 10 min, if required, oxygen can be delivered at the treating physician’s discretion from the hospital piped oxygen supply, alongside ongoing medical treatment of CSE.

### Criteria for discontinuing or modifying allocated interventions {11b}

Some situations of possible early termination of trial treatment are foreseen:

1. IMP inhalation is interrupted by a child becoming more aware of their circumstances (e.g. because of early termination of CSE), becoming distressed and pulling off the mask.
2. IMP inhalation has to be interrupted, e.g. to provide suction to clear secretions or vomit from the airway.
3. Occasionally treating teams will institute temporary, manual ventilatory support as respiratory depression is an important complication of current treatment of CSE (particularly where administration of parenteral benzodiazepines has occurred). Where possible, these devices will be attached directly to a cylinder supply so that IMP can continue. However, some alternatives can only be driven via an anaesthetic machine and so cannot be attached directly to an IMP cylinder.

In these and in all similar circumstances, data collection will continue and reasons for discontinuation or modification of treatment will be collected. Data will be analysed on an intention to treat basis. Given the very short duration of the intervention, intervention discontinuation at participant/parent/representative request is not anticipated.

### Strategies to improve adherence to interventions {11c}

It is not applicable due to the very short (10 min) duration of the intervention administered within the ED setting.

### Relevant concomitant care permitted or prohibited during the trial {11d}

There are no restrictions in relation to concomitant medication or treatments. The following data on concomitant medication will be recorded on the case report form (CRF):

- Benzodiazepines administered prior to randomisation (i.e. pre-admission)
- Pre-admission epilepsy medication
- The use of other first-line and second-line drugs to treat the seizure

### Provisions for post-trial care {30}

Participants receive trial IMP gas for 10 min. Upon completion, all ongoing care is as per clinical judgement without restriction. There are no special compensation arrangements. However, if a participant is harmed due to someone’s negligence, then they may have grounds for a legal action for compensation against the NHS Trust where they are being treated but they may have to pay for their legal costs.

## Outcomes {12}

### Primary outcome

The primary outcome is the success of first-line treatment of CSE (i.e. child did not need to receive second-line or rescue therapy). Success of first-line treatment is defined as not requiring intravenous phenytoin, phenobarbital, valproate, levetiracetam, or rapid sequence induction of general anaesthesia (paraldehyde is considered a first-line medication).

### Secondary outcomes

- Seizure activity visible at 5 min and 15 min post commencement of inhalation
- Need for rapid sequence induction (RSI) with thiopentone or another agent (e.g. propofol) due to ongoing CSE
- Need to be admitted to critical care (PICU) or high dependency unit
- Seizure recurrence within 24 h
- Serious adverse events and reactions
- 30-day mortality

**Table:** 

Objectives	Outcome measures	Timepoint(s) of evaluation
Effectiveness:		
Whether concurrent carbogen use increases response rates to first-line treatment of CSE	Success of first-line treatment of CSE (i.e. child did not need to receive second-line or rescue therapy as defined within the APLS guidelines or their personal treatment plan). Success of first-line treatment is defined as not requiring intravenous phenytoin, phenobarbital, valproate, levetiracetam, or RSI	24 h
	Seizure activity visible at 5 min and 15 min post commencement of inhalation	At 5 min and 15 min post commencement of inhalation
	Need for rapid sequence induction (RSI) with thiopentone or another agent (e.g. propofol) due to ongoing CSE	24 h
	Need to be admitted to critical care or high dependency unit	24 h
	Seizure recurrence within 24 h	24 h
Safety:		
Ascertain the safety of carbogen	Serious adverse events and reactions	Up to 30 days post randomisation with an onset within 24 hof inhalation
	Mortality within 30 days of enrolment	30 days

### Participant timeline {13}


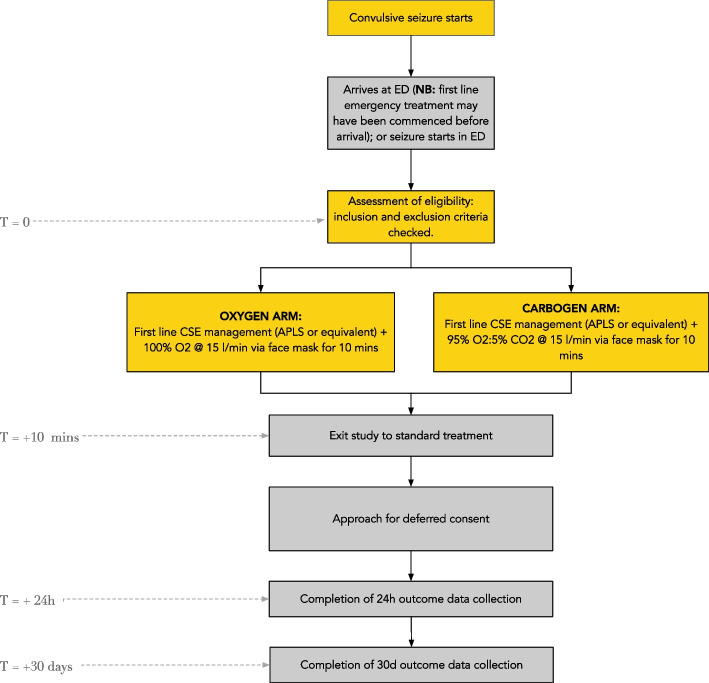



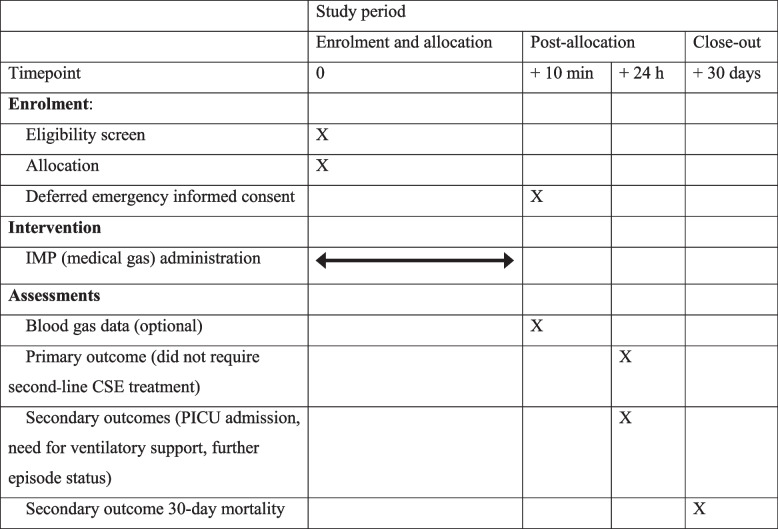


### Sample size {14}

The CRESCENT trial aims to recruit 424 participants. The trial will include a 12-month internal pilot with stop/go criteria assessing recruitment, consent rates, and data completeness. Sites open and recruiting for a minimum of 3 months each will be included.

The sample size calculations were informed by the screening data from EcLiPSE. Of 1432 screenings, 286 went on to receive second-line treatment, suggesting an 80% success rate of first-line intervention [8]. However, practices varied at sites with some only screening patients once progression to second-line intervention became increasingly likely. Restricting to those sites where screening data included all children presenting with a seizure, a success rate of 85% was observed for first-line treatment.

Using a control group success rate for first-line treatment of 85%, we aim to detect a 10% increase using a group sequential design with one interim analysis planned following observation of the primary outcome for 50% of the recruitment target. Sample size calculations were undertaken in NQuery using the O’Brien-Fleming spending function to determine the upper efficacy test boundary (0.025) and the futility boundary (non-binding).

Bounds for efficacy to reject the null hypothesis are 2.963 and 1.97 respectively for the interim and final analysis with the futility boundary for acceptance of the null hypothesis at the interim analysis being 0.254. This requires 191 participants per group for the final analysis to achieve 5% two-sided type 1 and 10% type II error rates.

As with EcLiPSE, data completeness is expected to be exceptionally high (99%); however, the overall sample size target is inflated to allow for a maximum of 10% loss to follow-up.

### Recruitment {15}

A very high proportion of eligible children will be enrolled due to the close alignment with standard care, minimisation of data to be collected, and use of deferred consent. The main threat to recruitment is ensuring children are seen on arrival by staff approved on the delegation log to assess eligibility and initiate treatment, in the face of rapid staff turnover. Eight sites have been opened initially based on recruitment data from EcLiPSE, but if necessary additional sites may be opened. PIs at all sites are active members of the PERUKI network.

## Assignment of interventions: allocation

### Sequence generation {16a}

A computer-generated random allocation sequence with random variable block size will be generated by LCTC and supplied to the IMP manufacturer (BOC Group) to inform the sequence of cylinder content fillings (1:1 ratio). Cylinders are used in numbered sequence order. The emergency nature of the project limits the potential to stratify the randomisation process.

### Concealment mechanism {16b}

Identical, sequentially numbered cylinders. See the “Sequence generation {16a}” section.

### Implementation {16c}

See the “Sequence generation {16a}” section. Participants will be enrolled by appropriately qualified health professionals with the experience to diagnose CSE, consider and exclude differential diagnoses, and initiate emergency treatment. Although this will typically be a medically qualified doctor, the protocol permits advanced nurse practitioners (ANPs), advanced clinical practitioners (ACPs), and equivalently qualified professionals to perform this role. These individuals must already perform these roles as part of their standard clinical duties and their hospital’s standard operating procedures.

## Assignment of interventions: blinding

### Who will be blinded {17a}

All staff at participating sites, caregivers and participants are blinded. All outcome assessments are conducted at sites. The statistical team will not be blind. Requests for the allocations by the statistical team will be controlled. The decision not to blind the statistical team was informed by the trials risk assessment, including the objective outcome measures and recent research [27].

### Procedure for unblinding if needed {17b}

There is no emergency unblinding process for participants randomised into the CRESCENT trial. The composition of gases in lungs and blood re-equilibrates extremely rapidly (within seconds) of reversion to spontaneous or assisted ventilation in air or another inhaled gas mixture. For this reason, there is no need to unblind a participant’s allocation contemporaneously. Knowledge of the IMP received would not change the medical management (which would continue according to APLS principles), and any unblinding process would introduce unnecessary delays into the management of an emergency, especially as the CTIMP is delivered for a maximum of 10 min.

## Data collection and management

### Plans for assessment and collection of outcomes {18a}

Participant CRF folders will be provided to sites for local completion by members of the research team trained and delegated the duty. All staff delegated to these duties will undergo formal training on how to do so. Trial staff named at each site will enter data from source documents corresponding to a participant’s visit onto the relevant CRF in the participant’s folder. The CRF is the primary data collection instrument for the study, so all data requested on the CRF must be recorded and all missing data must be explained. A copy of all CRFs will be retained at site with the original wet-ink editions transferred and held at the LCTC. Any corrections will be made in accordance with the principles outlined in Good Clinical Practice in Research.

### Plans to promote participant retention and complete follow-up {18b}

All subsequent follow-up data is obtained from routine hospital medical records with no trial-specific follow-up. Data collection will be completed for all participants including those in whom IMP was discontinued early for any of the reasons outlined in the “Criteria for discontinuing or modifying allocated interventions {11b}” section.

### Data management {19}

Date(s) of informed consent and assent processes (including date of provision of patient information, randomisation number and the fact that the patient is participating in a clinical trial and stating possible treatment arms) will be added to the patient’s medical record chronologically.

Trial data will be collected on paper case report forms (CRFs) by site clinical or research staff who have been trained and delegated to do so. The CRF is the primary data collection instrument for the trial; all data must be recorded, and all missing data must be explained. Data will be entered into a validated database by the LCTC data manager in accordance with the procedures described within the trial Data Entry & Cleaning Manual. During data processing, automated and manual checks are undertaken for missing or unusual values (range checks) and for consistency within participants over time. Other data checks relevant to patient rights and safety are regularly performed as per LCTC processes. Any suspect data will be returned to the site in the form of data queries. Data query forms are produced at LCTC from the trial database and sent to a named individual (as listed on the site delegation log). Sites respond to queries providing an explanation/resolution to discrepancies and return the data query forms to LCTC. The forms are filed along with the appropriate CRFs, and the appropriate corrections made on the database.

The database security, user access, and privileges are all handled by the Information Systems Team in LCTC. Database access privileges are allocated according to delegated responsibilities. The data are backed up in such a way as to allow for restoration in the event of hardware failure.

All paper-based study data and documentation for the trial are stored in the Trial Master File (TMF). This includes all complete CRFs and associated data query forms. LCTC will securely store the TMF for a period of 25 years from end of trial. The database will remain on the server for 25 years from end of trial.

### Confidentiality {27}

All data collected in this trial will be handled and processed confidentially and securely and in accordance with applicable data protection legislation.

Personal identifiable data is only collected within the consent process and held separately. CRFs will be labelled with a unique trial randomisation number. CRFs received at LCTC contain non-identifiable data and are anonymous until the corresponding consent/assent form is received. Verification that appropriate informed consent is obtained will be enabled by the provision of copies of participant’s signed informed consent and assent forms being supplied to LCTC by recruiting sites. This transfer of identifiable data is disclosed in the patient information sheet and consent form (PISC).

Where consent is not obtained for a participant, a minimum data set will be provided to LCTC. The minimum data set will not include any personal identifiers and will therefore be anonymous for the purposes of the recipient (LCTC) in accordance with ICO guidance [28] and compliance with the Common Law Duty of Confidentiality.

Where consent is obtained, data is considered identifiable (i.e. Personal Data under data protection legislation)—and the consent process obtains permissions for disclosure under Common Law Duty of Confidentiality purposes.

The principle of data minimisation has been used throughout the development of the CRESCENT CRFs. Data (including special category) will only be collected, used, and stored if necessary for the trial (e.g. evidencing provision of consent, for data management and central monitoring, statistical analysis, regulatory reporting, etc.).

### Plans for collection, laboratory evaluation, and storage of biological specimens for genetic or molecular analysis in this trial/future use {33}

Not applicable: no samples are being collected.

## Statistical methods

### Statistical methods for primary and secondary outcomes {20a}

The primary analysis will analyse all participants in the group to which they were randomly allocated. A 5% level of statistical significance will be used throughout, and all results will be presented with 95% confidence intervals (95% confidence interval for the primary outcome calculated to allow for interim analysis alpha-spend). A detailed statistical analysis plan will be written prior to the first meeting of the IDSMC in which un-blinded data are required. The statistical analysis plan will be submitted for publication and made publicly available [29].

The primary outcome is success of first-line treatment of CSE (i.e. child did not need to receive second-line or rescue therapy as defined within the APLS guidelines or their personal treatment plan). Success of first-line treatment is defined as not requiring intravenous phenytoin, phenobarbital, valproate, levetiracetam, or RSI. Instead, the primary analysis will be by logistic regression and will include known prespecified important prognostic indicators. The primary outcome will be analysed using logistic regression and will adjust for these prespecified variables.

All secondary outcomes are binary outcomes which will be analysed as per the primary outcome. Serious and adverse events and reactions are expected to be of low frequency and will be summarised using descriptive statistics only.

### Interim analyses {21b}

CRESCENT is a group sequential design with one interim analysis planned following observation of the primary outcome for 50% of the recruitment target. The boundaries for the interim analysis are described within the “Sample size {14}” section.

The timing of interim analyses may be altered at the discretion of the IDSMC. The results of this and any other interim analyses will be reviewed by the IDSMC who will make recommendations to the trial steering committee regarding trial continuation and modification. A decision to discontinue recruitment, in all patients or in selected subgroups, will be made only if the result is likely to convince a broad range of clinicians including participants in the trial and the general clinical community.

### Methods for additional analyses (e.g. subgroup analyses) {20b}

Two prespecified, exploratory analyses will be conducted. We will examine whether there is evidence of greater response rates to carbogen in children whose first blood pH is more alkalotic and in children deemed to have had a febrile CSE episode.

### Methods in analysis to handle protocol non-adherence and any statistical methods to handle missing data {20c}

Due to the very short duration of the protocol, no significant non-adherence is anticipated.

### Plans to give access to the full protocol, participant-level data, and statistical code {31c}

The full trial protocol is available on the funder’s web-page (https://fundingawards.nihr.ac.uk/award/NIHR129875). At the end of the trial, after the primary results have been published, anonymised individual participant data and associated documentation (e.g. protocol, annotated blank CRF) will be prepared in order to be shared with external researchers. All requests for access to the trial data will be discussed with the sponsor in accordance with the sponsor policy on data sharing. Statistical code will not be shared.

## Oversight and monitoring

### Composition of the coordinating centre and trial steering committee {5d}

The trial management group (TMG) is comprised of the chief investigator, co-applicants, sponsor representative, sponsor pharmacy representative(s), patient representative(s), and members of the LCTC. The TMG are responsible for the day-to-day running and management of the trial. The TMG will meet as defined in their terms of reference.

The trial steering committee consists of an independent chairperson (biostatistician/trialist), independent experts in the fields of paediatric emergency medicine and epilepsy, parent representatives, and the chief investigator. The role of the TSC is to provide oversight for the trial and provide advice through its independent chair. The TSC will consider recommendations of the independent data and safety monitoring committee (IDSMC). The TSC judgement on IDSMC recommendations for the continuation or amendment of the trial will be communicated to the trial sponsor and funder. The TSC will meet throughout the trial (at least annually) in accordance with their terms of reference/charter.

The composition and membership of the TSC has been confirmed by NIHR EME as funder.

### Composition of the data monitoring committee, its role and reporting structure {21a}

The IDSMC consists of an independent chairperson (biostatistician/trialist) plus two independent members, experts in the fields of paediatrics and paediatric epilepsy.

The composition and membership of the IDSMC has been confirmed by NIHR EME as funder.

The IDSMC will receive and review monitoring reports and interim analyses for the trial and provide recommendations on the conduct of the trial to the trial steering committee in accordance with their terms of reference.

### Adverse event reporting and harms {22}

Active monitoring of safety events experienced by trial participants will be for 30 days from randomisation but limited to only serious events or serious reactions with an onset within 24 h of the initial inhalation. This is justified on the basis of the extremely rapid re-equilibration of blood gas composition following alterations to inspired gas mixture (i.e. there are no plausible grounds for later onset events to be related to trial participation).

Safety will be assessed by the principal investigator (PI) or delegated research staff at site monitoring and reporting all serious adverse events and serious reactions from randomisation during this period. Note that hospitalisation and/or PICU admission for treatment of the episode of CSE per se (including rapid sequence induction of general anaesthesia) and/or support for respiratory depression associated with its treatment need not be regarded as serious if, in the view of the treating physician, the clinical course is typical of CSE and its management. The decision to regard hospitalisation and/or PICU admission as indicative of a ‘serious’ adverse event or adverse reaction (AE/AR) is at treating physician’s discretion. If a recruited participant is transferred to another hospital, then follow-up with the accepting hospital will be completed to ensure that the data recorded are accurate. Thirty-day mortality will be ascertained by sites by reviewing the medical notes. Expedited and annual reporting of safety data will be submitted to REC and the MHRA in compliance with UK regulatory requirements.

Female participants found to have been pregnant at enrolment will be followed up until the outcome of the birth.

The monitoring plan for CRESCENT has been informed by the trials risk assessment.

### Frequency and plans for auditing trial conduct {23}

LCTC internal and sponsor external audits are conducted as a systemic examination of trial-related activities, systems, and documents to determine whether the evaluated trial-related activities were conducted and the data recorded, analysed, and accurately reported according to the protocol, organisational standard operating procedures, GCP, and the applicable regulatory guidance. Internal audits are conducted at the LCTC by a dedicated Quality & Regulatory Assurance Department who do not have daily operational input into the conduct of CRESCENT. External audits are conducted by a dedicated GCP audit department who are independent from the sponsor operational management group. The LCTC and sponsor adopt a risk-based approach to performing internal and external audits regarding frequency and prioritisation. This approach considers inherent study risks, previous audit or inspections findings, and any identified compliance issues such as potential serious breaches of GCP and protocol and breaches of data protection principles. The results of all audits are shared between the LCTC and sponsor for the benefit of continuous improvement in CRESCENT. As with all clinical trials within the UK, CRESCENT is also subject to audits by UK regulatory authorities who maintain complete independence.

In order to perform their role effectively, the trial manager and persons involved in quality assurance and inspection may need direct access to primary data, e.g. patient medical records, laboratory reports, appointment books. Since this affects the participant’s confidentiality, this fact is included on the PISC. In agreeing to participate in this study, a PI grants permission to the sponsor (or designee) and appropriate regulatory authorities to conduct on-site monitoring and/or auditing of all appropriate study documentation.

### Plans for communicating important protocol amendments to relevant parties (e.g. trial participants, ethical committees) {25}

The protocol, participant information, and consent/assent materials and any proposed public-facing material will be submitted to the Research Ethics Committee (REC), the Health Research Authority (HRA), and the Medicines and Healthcare products Regulatory Agency (MHRA) and trial sites for approval. Any substantial amendments to the original approved documents will be submitted and, where necessary, approved by the above parties before use. Any change in trial design which may require consideration of re-consent of participants will also be assessed at each amendment. A data privacy statement will be maintained on the LCTC website and updated accordingly.

Protocol amendments will also be submitted for approval to the trial funders. All protocol versions may be accessed via the funders trial webpage (https://fundingawards.nihr.ac.uk/award/NIHR129875).

### Dissemination plans {31a}

Training materials will be developed and made available to participating sites covering both ED enrolment (simulation scenarios and videos) and around approach for deferred consent.

Trial results will be disseminated to healthcare professionals through peer-reviewed publication and presentation at scientific congresses. If trial results support the use of carbogen in management of CSE, this will be disseminated to epilepsy and emergency medicine communities via PERUKI and international paediatric emergency medicine networks. Implications for further research into use in pre-hospital settings will also be discussed with guideline developers and other key stakeholders in pre-hospital care such as Resuscitation Council UK. Results will be disseminated to the general public via social media, plain English summaries, and infographics, etc., including a lay summary submitted to HRA as part of the final results submission. Trial results will be communicated in plain English to participants who elected and consented to receive this information.

## Discussion

The main threat to recruitment is the 24/7 nature of presentations to EDs in CSE and the need to ensure that such children will be first seen by appropriately trained, delegated clinicians at all sites at all times and to maintain this in the face of shift-working and regular changes of ‘house’ as junior doctors rotate through short-term placements. An on-line delegation log is used to facilitate this and associate principal investigators (API) at sites will assist in maintaining its currency.

To ensure accurate and high-quality data every effort has been made to minimise the data collected with longer term follow-up post discharge being completed using routinely collected data. Data completeness and quality will be monitored throughout the trial.

Due to the nature of paediatric CSE, for many families participating in the trial, this will be a first-ever seizure event (often in a young child). For others, it will be the latest of many episodes. The trial has greatly benefitted from the contributions of the patient and public involvement member of the TMG who is in the latter group. She has taken an active role in trial design and will do so again during the dissemination of trial results. We have also consulted with parents of healthy young children as representatives of the first group.

## Trial status

Protocol V3.0 17/11/2022.

First patient recruited 22 June 2023.

Anticipated end of recruitment 31 December 2025.

## Abbreviations

APLS: Advanced Paediatric Life Support (resuscitation guidelines)
BOC: British Oxygen Company (supplier of medical gases)
CRF: Case report form
CSE: Convulsive status epilepticus (see below for lay definition)
CTU: Clinical trials unit
EcLiPSE: Emergency treatment with Levetiracetam or Phenytoin in convulsive Status Epilepticus (previous successfully completed clinical trial)
EudraCT: European Union Drug Regulating Authorities Clinical Trials Database
ED: Emergency department (= accident and emergency department)
FiCO2: Proportion of carbon dioxide in inspired gas
GABA: Gamma-amino butyric acid
ICU: Intensive care unit
IDSMC: Independent data and safety monitoring committee
IMP: Investigational medicinal product
ISRCTN: International Standard Randomised Controlled Trials Number
ITT: Intention-to-treat
LCTC: Liverpool Clinical Trials Centre
MHRA: The Medicines and Healthcare products Regulatory Agency
MGDS: Medical Gas Data Sheet
MRS: Magnetic resonance spectroscopy
NHS: National Health Service
NIHR EME: National Institute for Health Research Efficacy and Mechanism Evaluation (EME) programme
NUTH: Newcastle upon Tyne Hospitals NHS Foundation Trust (sponsor)
paCO2: Partial pressure of carbon dioxide in arterial blood
PI: Principal investigator
PICU: Paediatric intensive care unit
PISC: Patient information sheet and consent form
RCT: Randomised controlled trial
REC: Research ethics committee
RSI: Rapid sequence induction (of general anaesthesia)
TMG: Trial management group
TSC: Trial steering committee
